# A Novel Fused SiO_2_ and h-BN Modified Quartz Fiber/Benzoxazine Resin Ceramizable Composite with Excellent Flexural Strength and Ablation Resistance

**DOI:** 10.3390/polym15224430

**Published:** 2023-11-16

**Authors:** Zongyi Deng, Yunfei Lv, Minxian Shi, Zhixiong Huang, Wenchao Huang

**Affiliations:** 1 Key Laboratory of Advanced Technology for Specially Functional Materials, Ministry of Education, Wuhan University of Technology, Wuhan 430070, China; zong_yi_deng@whut.edu.cn (Z.D.); minxianshi@whut.edu.cn (M.S.); zhixiongh@whut.edu.cn (Z.H.); 2 Hubei Longzhong Laboratory, Xiangyang 441000, China; 3 Beijing FRP Institute Composite Materials Co., Ltd., Beijing 102101, China; xiaochi8105@126.com

**Keywords:** benzoxazine resin, ceramizable composite, thermal stability, flexural strength, ablation resistance, thermal protective barrier

## Abstract

Hypersonic vehicles encounter hostile service environments of thermal/mechanical/chemical coupling, so thermal protection materials are crucial and essential. Ceramizable composites have recently attracted intensive interest due to their ability to provide large-area thermal protection for hypersonic vehicles. In this work, a novel ceramizable composite of quartz fiber/benzoxazine resin modified with fused SiO_2_ and h-BN was fabricated using a prepreg compression molding technique. The effects of the fused SiO_2_ and h-BN contents on the thermal, mechanical, and ablative properties of the ceramizable composite were systematically investigated. The ceramizable composite with an optimized amount of fused SiO_2_ and h-BN exhibited superb thermal stability, with a peak degradation temperature and residue yield at 1400 °C of 533.2 °C and 71.5%, respectively. Moreover, the modified ceramizable composite exhibited excellent load-bearing capacity with a flexural strength of 402.2 MPa and superior ablation resistance with a linear ablation rate of 0.0147 mm/s at a heat flux of 4.2 MW/m^2^, which was significantly better than the pristine quartz fiber/benzoxazine resin composite. In addition, possible ablation mechanisms were revealed based on the microstructure analysis, phase transformation, chemical bonding states, and the degree of graphitization of the ceramized products. The readily oxidized pyrolytic carbon (PyC) and the SiO_2_ with a relatively low melting point were converted in situ into refractory carbide. Thus, a robust thermal protective barrier with SiC as the skeleton and borosilicate glass as the matrix protected the composite from severe thermochemical erosion and thermomechanical denudation.

## 1. Introduction

Hypersonic space shuttles and vehicles have attracted widespread attention recently due to their excellent maneuverability. However, hypersonic vehicles face extremely harsh service environments when flying in or crossing the atmosphere due to the severe aerodynamic heating phenomenon [1,2,3]. The harsh environment leads to not only thermochemical erosion caused by oxidation but also thermomechanical denudation caused by high-temperature and high-speed airflow erosion, and enormous thermal stress generated by instantaneous temperature rise [4,5]. In particular, with the development of hypersonic aircraft with high Mach numbers and long endurance, aerodynamic heating has become much more severe. Thus, thermal protection materials (TPMs) are regarded as key components in hypersonic vehicles [6,7,8]. According to the thermal protection mechanisms, TPMs can be divided into heat sink-, transpiration cooling-, radiation-, and ablation-type TPMs [9,10,11].

Heat sink-type TPMs are an essential type of TPMs, which utilize the material’s capacity to absorb heat. They typically use metals with high specific heat capacity, such as tungsten and molybdenum, which inherently have the drawback of high density [3,10]. Moreover, due to the upper limit of the materials’ endothermic energy storage, heat sink-type TPMs are only suitable for short-term thermal protection [3]. Transpiration cooling-type TPMs absorb heat via chemical and physical processes, such as the decomposition or gasification of cooling agents. The advantage of this type of TPM is that it maintains a good aerodynamic shape, and the yielded gas film is also conducive to resisting the erosion of particles in space. However, the complex preparation process and high cost limit large-scale applications [3,10]. Radiation-type TPMs can dissipate heat via the radiation of high-emissivity materials, such as silicides, carbides, and borides of transition metals [8,12]. They exhibit excellent heat resistance, oxidation resistance, and ablation resistance under hostile service environments and can be used as long-term thermal protection materials in hypersonic vehicles [4,13]. However, they exhibit high density, complex preparation process, high manufacturing temperature (typically above 2000 °C), long preparation cycle, and high price. Therefore, they are only applied in local ultra-high-temperature parts, such as the nose tip, sharp leading edge of the body, and inlet of the engine, but fail to provide large-area thermal protection for hypersonic vehicles [3,8]. Ablation-type TPMs dissipate heat by sacrificing themselves (such as pyrolysis, melting, sublimation, and other physical and chemical reactions) and lose part of their own mass [5,14]. They are the most widely used TPM thermal protection materials, especially for missiles and spacecraft. Ablation-type TPMs can be mainly divided into carbon-based ablation materials and silicon-based ablative materials according to the material category [5,10,15].

Carbon/carbon composites (C/C) and carbon fiber/phenolic resin composites (CF/Ph) are typical representatives of carbon-based ablation materials that have high specific strength and modulus at room temperature [16,17]. However, as a carbon material, C/C inevitably has the disadvantage of being easily oxidized. Surface modification can enhance the oxidation and ablation resistance of C/C composite materials, but the complex preparation process and long production cycle limit the potential application in large-area thermal protection for hypersonic vehicles [8,18,19]. Similarly, CF/Ph is also prone to oxidation failure. The oxidation resistance and ablation resistance of CF/Ph could be significantly improved via matrix modification and fiber-coating strategies [5,8,20]. For example, Chang et al. reported an Al_2_O_3f_-CF/Ph modified with HfB_2_ and SiB_6_ that achieves a linear ablation rate (LAR) of 0.06 mm/s [21]. Yue et al. fabricated a mesophase pitch-modified CF/Ph, and its LAR was as low as 0.0082 mm/s [22]. In our previous work, MoSi_2_ and B_4_C were incorporated into CF/Ph to develop a new ceramizable composite exhibiting satisfactory ablation resistance with an LAR of 0.013 mm/s [20]. Furthermore, a Ti_3_SiC_2_-modified Al-coated carbon fiber/boron phenolic resin ceramizable composite was prepared by combining matrix modification and fiber-coating strategies. The resulting ceramizable composite exhibited excellent ablation resistance, and its LAR was as low as 0.00853 mm/s [23]. However, due to the high thermal conductivity of the carbon fibers, the insulation performance of CF/Ph was poor.

High-silica fiber/phenolic resin composites (HSF/Ph) and quartz fiber/phenolic resin composites (QF/Ph) are typical silicon-based ablation materials, and they have much lower thermal conductivity and better heat insulation performance [8,24]. Polymer matrix composites not only have the advantages of high specific strength and modulus at room temperature but they can also be easily manufactured into a large-scale size via a simple and facile molding process [10,25]. However, traditional HSF/Ph and QF/Ph have poor heat resistance and ablation resistance, making them unsuitable for ultra-high temperature, long-endurance, and large-area thermal protection in hypersonic vehicles [8]. Many attempts have been made to improve the thermal protection properties of HSF/Ph and QF/Ph, so as to meet the increasingly stringent service requirements of hypersonic vehicles [10,23]. For example, Wang et al. reported an HSF/Ph modified with nano-Al_2_O_3_, and its LAR was 0.072 mm/s, which was 15.3% lower than that of its unmodified counterpart [26]. Wang et al. prepared an HSF/Ph modified with B_4_C and talc; its compressive strength obviously decreased at 600 °C, but increased at 800–1000 °C [27]. Yan et al. fabricated a needled quartz fiber felt/phenolic resin composite prepared via vacuum pressure impregnation processing, and its LAR was 0.0227 mm/s [28]. Wang et al. reported a lightweight quartz felt/phenolic aerogel composite modified with ZrB_2_, SiB_6_, SiO_2,_ and Al_2_O_3_, and its LAR was 0.017 mm/s [29]. In our previous work, B_4_C and ZrSi_2_ were used to modify QF/Ph, and their thermal oxidation and compressive failure behaviors were significantly improved [30].

Benzoxazine resin, a novel high-performance phenolic resin, has lower curing shrinkage, higher residue yield, and superior mechanical properties than other traditional phenolic resin [31,32,33,34,35,36]. However, few studies investigated the ablation resistance of quartz fiber/benzoxazine resin composites. In this work, a novel quartz fiber/benzoxazine resin ceramizable composite modified with fused SiO_2_ and h-BN was developed. The effects of the fused SiO_2_ and h-BN on the density, thermal conductivity, thermal degradation behavior, flexural strength, and ablation resistance of the ceramizable composite were systematically investigated. Furthermore, the ablation mechanism of this newly reported composite was investigated.

## 2. Materials and Methods

### 2.1. Raw Materials

Benzoxazine resin (CB6800) was supplied by Chengdu Coryes Polymer Science & Technology Co., Ltd. (Chengdu, China). Acetone was bought from Sinopharm Chemical Reagent Co., Ltd. (Shanghai, China). Quartz fiber plain cloth (B type, 142 gsm) was purchased from Hubei Feilihua Quartz Glass Co., Ltd. (Jingzhou, China). Fused SiO_2_ particles (1 μm, 99.5%) and h-BN particles (1 μm, 99.5%) were bought from Shanghai Buwei Applied Materials Technology Co., Ltd. (Shanghai, China). and Forsman Scientific (Beijing, China) Co., Ltd., respectively (Beijing, China). The details of the used materials are summarized in Table S1.

### 2.2. Preparation of Composite

**Figure 1:**
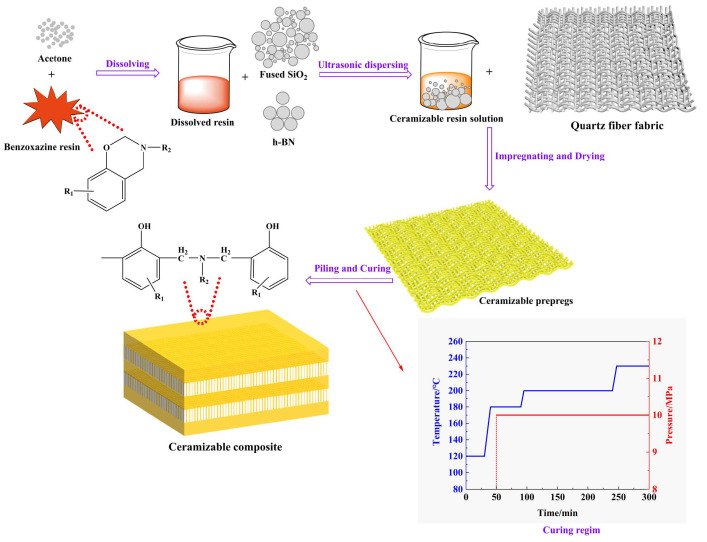
Schematic diagram of the preparation process of ceramizable composites.

Quartz fiber/benzoxazine resin composites incorporating different contents of fused SiO_2_ and h-BN (Table 1) were prepared via a prepreg compression molding (PCM) technique, as illustrated in Figure 1. First, the as-received benzoxazine resin paste was dissolved in acetone with a ratio of 1:1 in an ultrasonic water bath at 50 °C for 1 h to form a homogeneous benzoxazine resin solution. Second, fused SiO_2_ and h-BN were slowly added to the benzoxazine resin solution and then stirred at 800 rpm for 10 min, followed by an ultrasonic dispersion treatment for 15 min to generate a well-dispersed ceramizable resin solution. The quartz fiber fabric was impregnated with ceramizable resin solution and naturally air-dried at 30 °C for 7 days to remove acetone. The prepregs were piled, molded, and cured in a plate vulcanizing machine via a PCM technique following the curing process illustrated in Figure 1.

### 2.3. Oxyacetylene Ablation Test

**Figure 2:**
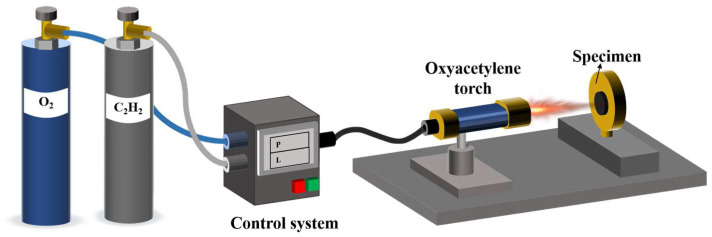
Schematic diagram of the oxyacetylene ablation test of the ceramizable composites.

In order to investigate the thermal protection performance, the samples were exposed to an oxyacetylene torch and ablated for 15 s following the Chinese standard GJB 323A-96 [37] (Figure 2). The test parameters are displayed in Table 2. The linear and mass ablation rates were calculated using the following formulas, respectively:

$$\mathrm{Linear}\;\mathrm{ablation}\;\mathrm{rate}\;(\mathrm{LAR})=\frac{\left(l_{1}-l_{2}\right)}{\Delta t}$$

$$\mathrm{Mass}\;\mathrm{ablation}\;\mathrm{rate}\;(\mathrm{MAR})=\frac{\left(m_{1}-m_{2}\right)}{\Delta t}$$

where l_1_ and l_2_ are denoted as the initial depth of samples before ablation and the ultimate depth of samples after ablation (mm), respectively; m_1_ and m_2_ stand for the initial mass of samples before ablation and the ultimate mass of samples after ablation (mm), respectively; Δt represents ablation time (s) and represents 15 s in this study.

### 2.4. Characterizations

Group changes in the samples were recorded using a Fourier-transformed infrared spectrometer (FT−IR, Nexus, Thermo Nicolet Co., Ltd., Waltham, MA, USA) in the range of 4000 to 400 cm^−1^ with a step size of 4 cm^−1^. The density of the composites was measured using a geometric method following the Chinese recommended standard GB/T1463-2005 [38], and the mean value of five samples was calculated. Thermal conductivity of the composites was measured using a thermal constant analyzer (TPS250S, Hot Disk AB IOC., Goteborg, Sweden), whereas thermal stability was analyzed using a comprehensive thermal analyzer (TG-DTG, STA449F3, NETZSCH Instruments Inc., Selb, Germany) from room temperature to 1400 °C with a heating rate of 10 °C/min in an ambient atmosphere. Evolved gas analysis was carried out via a gas chromatograph coupled with a mass spectrometer (Py-GC/MS, Agilent 6890N/5975, Santa Clara, CA, USA) at 500 °C in an air atmosphere using pyrolysis mode. Nylon 6/6, Kraton, and Polyethylene were used as standards for the chromatography calibration. A universal mechanical testing machine (RGM-2100, Shenzhen Reger Instrument Co., Ltd., Shenzhen, China) was adopted to test the flexural strength of the composites pre- and post-ablation statically following GB/T 1449-2005 [39]. A scanning electron microscope (SEM, MIRA LMS, Tescan Group, Brno, Czech) coupled with an energy-dispersive X-ray spectrometer (EDS, Oxford Instruments, Oxford, UK) was used to observe the morphology of the samples. A powder X-ray diffractometer (XRD, D8 advance, Bruker Corporation, Karlsruhe, Germany) was utilized to characterize the crystal structures of the samples at 10–80° with an angular scanning rate of 5 °/min. The chemical bonding states of the products were characterized using an X-ray photoelectron spectrometer (XPS, K-Alpha, Thermo Fisher Scientific, Waltham, MA, USA). The pass energy of survey scans and high-resolution scans was 100 eV and 30 eV, respectively, whereas the step was 1.0 eV and 0.1 eV, respectively. Raman spectra were recorded using a Raman spectrometer (Raman, InVia, Renishaw, London, UK) with an excitation wavelength of 633 nm.

## 3. Results and Discussion

### 3.1. The Change in Chemical Structure during the Curing Process

**Figure 3:**
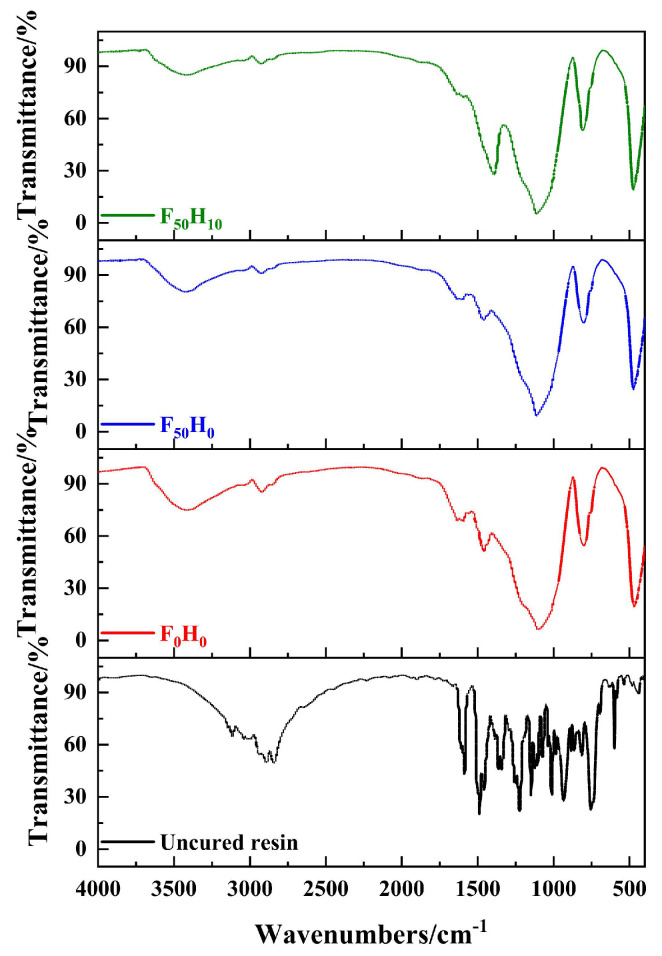
FT–IR spectra of the uncured resin and composites with different contents of fused SiO_2_ and h-BN.

The functional groups of the uncured resin and composites were identified via FT–IR (Figure 3). The characteristic vibration absorption peak (935 cm^−1^) of the oxazine ring disappeared after curing, indicating that the oxazine ring was fully opened during the curing process, as shown in Table 3. The absorption peaks of the symmetric and asymmetric stretching vibrations (1035 cm^−1^ and 1224 cm^−1^, respectively) of the ether bonds on the oxazine ring disappeared after curing. On the contrary, the blunt peak located at ~3420 cm^−1^ was observed after curing, corresponding to intermolecular hydrogen bonding, indicating the presence of phenolic hydroxyl groups in the molecular structure. It was confirmed that the oxazine ring was opened to form phenolic hydroxyl groups. In addition, the absorption peaks of the symmetric and asymmetric stretching vibrations (1148 cm^−1^ and 1363 cm^−1^, respectively) attributed to C-N-C bonds on the oxazine ring disappeared after curing. On the contrary, the strong absorption peak at ~1110 cm^−1^ after curing was attributed to the stretching vibration of the Mannich bridge bonds, which also confirmed the formation of a new C-N-C bond. The FT−IR results indicated that during the curing process, the benzoxazine resin underwent a ring-opening polymerization reaction.

### 3.2. Density, Thermal Conductivity, and Thermal Degradation Behavior of the Composites

**Figure 4:**
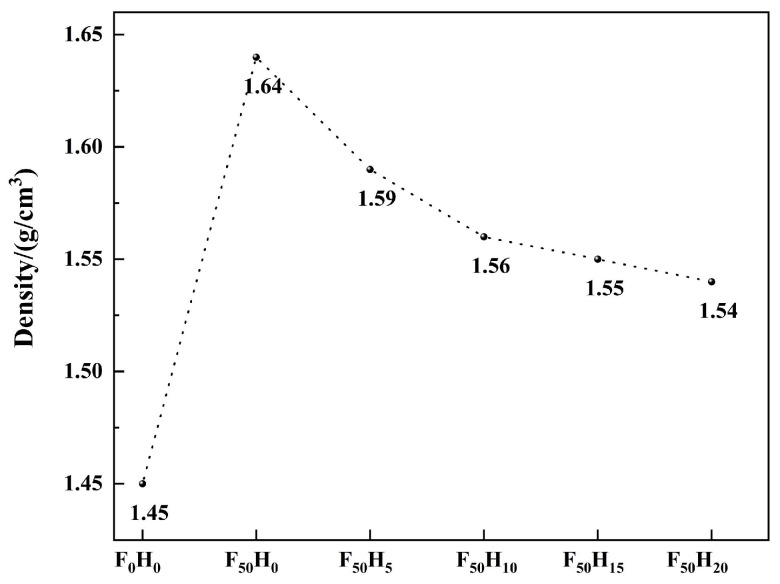
The density of the composites with different contents of fused SiO_2_ and h-BN.

The effect of the fused SiO_2_ and h-BN contents on the density, thermal conductivity, and thermal degradation behavior of the composites was investigated. The density of F_0_H_0_ was 1.45 g/cm^3^, and it notably increased to 1.64 g/cm^3^ when 50 phf of (part per hundred of fiber) fused SiO_2_ particles (F_50_H_0_) were added (Figure 4). Subsequently, the density decreased with an increase in the h-BN particles. As a result, the density of F_50_H_20_ was 1.54 g/cm^3^. The reasons for the significant increase in the density of the composites after the introduction of fused SiO_2_ were as follows. The density of fused SiO_2_ was much higher than that of the resin and fused SiO_2_ could also fill the pores caused by the escape of small molecules during the curing process, thereby increasing the density of the composites. In contrast, the h-BN particles were loose, so the density of the composites decreased with increasing h-BN particles.

**Figure 5:**
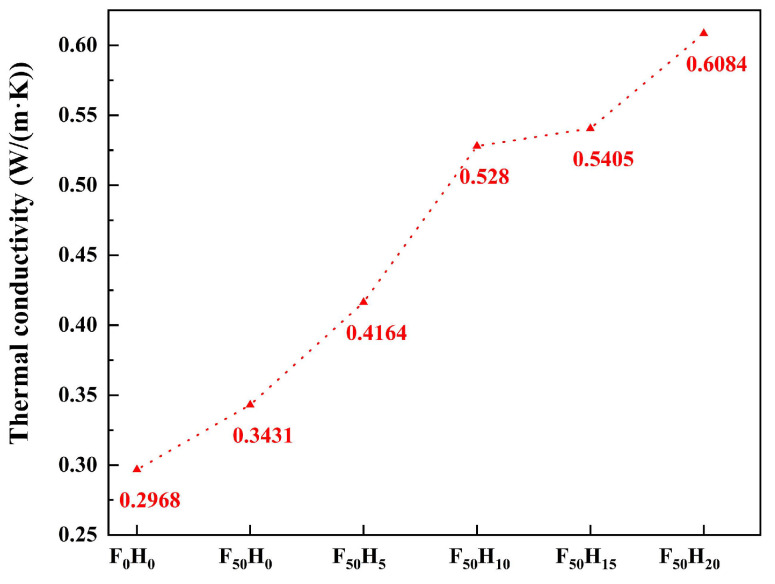
The thermal conductivity of the composites with different contents of fused SiO_2_ and h-BN.

In contrast, the thermal conductivity increased with increasing ceramizable fillers (Figure 5). The thermal conductivity of F_0_H_0_, F_50_H_0_, F_50_H_5_, F_50_H_10_, F_50_H_15,_ and F_50_H_20_ was 0.2968, 0.3431, 0.4164, 0.5280, 0.5405, and 0.6084 W/(m·K), respectively. It was easy to conclude that the thermal conductivity of the composites increased with increasing ceramizable fillers for the thermal conductivity network, and the channels were constructed gradually, which was beneficial to the phonon heat transfer process, especially when the h-BN particles themselves were typical materials with high thermal conductivity.

**Figure 6:**
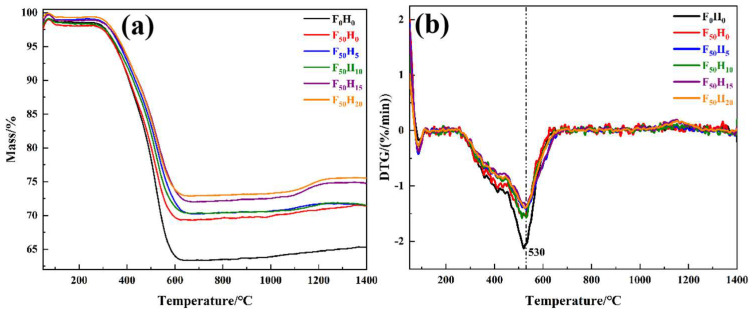
TG curves (**a**) and DTG curves (**b**) of the composites with different contents of fused SiO_2_ and h-BN.

**Figure 7:**
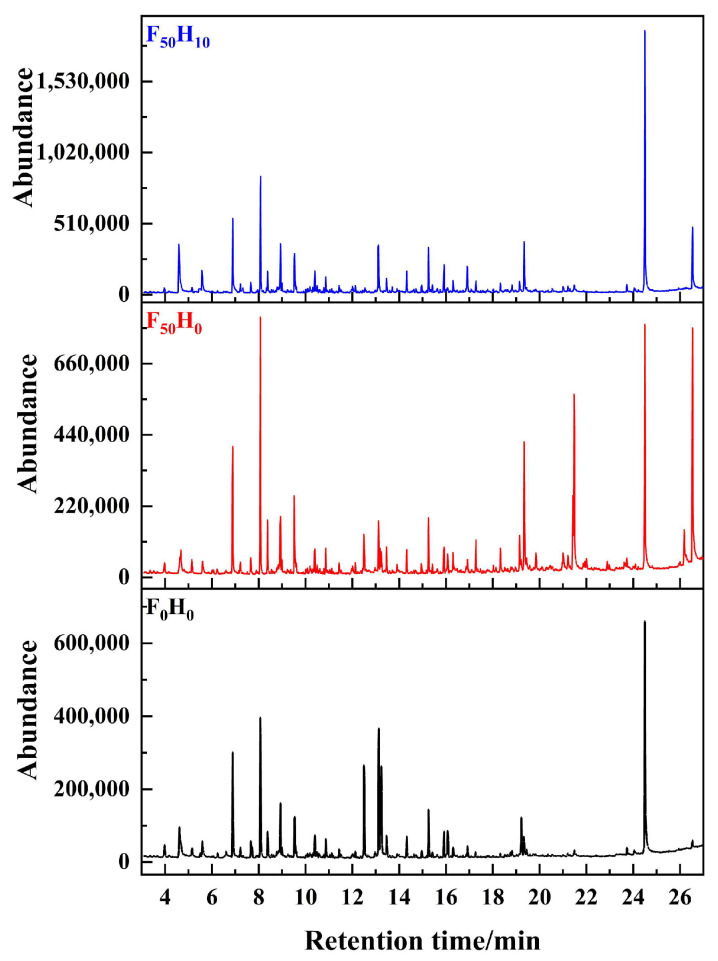
Total ion chromatograms of the evolved gas identified via Py-GC/MS.

The thermal degradation behaviors were investigated via TG-DTG and Py-GC/MS. As shown in Figure 6, there was no significant difference in the TG and DTG curves of the composites with different formulations. The above findings indicated that the ceramizable fillers did not affect the thermal degradation behaviors of the resin. The thermal degradation process of the composites could be divided into three stages. At first, a slight weight loss was observed below 300 °C, which was caused by the volatilization of the absorbed water. Following that, there was a violent weight loss between 300 and 600 °C, which was attributed to the pyrolysis of the resin. In addition, the detailed degradation products in the gas phase were identified via Py-GC/MS (Figure 7), and they are summarized in Tables S2–S4. Plenty of benzene derivatives were derived from the benzene skeleton of the resin, and nitrogen-containing compounds were derived from the identified oxazine structure, indicating that the backbone of the resin was destroyed. As the temperature increased above 600 °C, there was no significant change in the weight anymore. However, the residue yield of the samples exhibited an increase as the amount of ceramizable filler increased. Moreover, the thermal degradation temperature increased to higher values when more ceramizable fillers were introduced. The addition of ceramizable fillers can significantly improve the thermal stability of the composites and the oxidation resistance of the pyrolytic carbon (PyC).

### 3.3. Flexural Strength of the Composites

**Figure 8:**
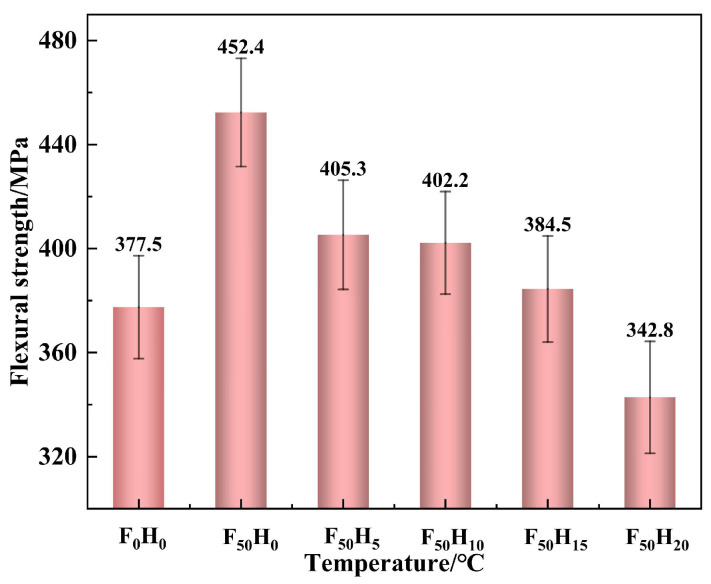
The flexural strength of the composites with different contents of fused SiO_2_ and h-BN.

**Figure 9:**
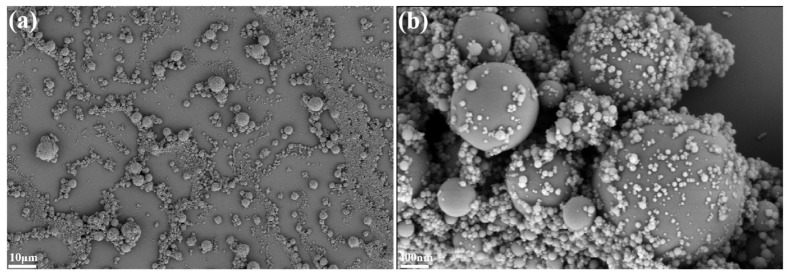
The SEM images of the fused SiO_2_ at low magnification (**a**) and at high magnification (**b**).

**Figure 10:**
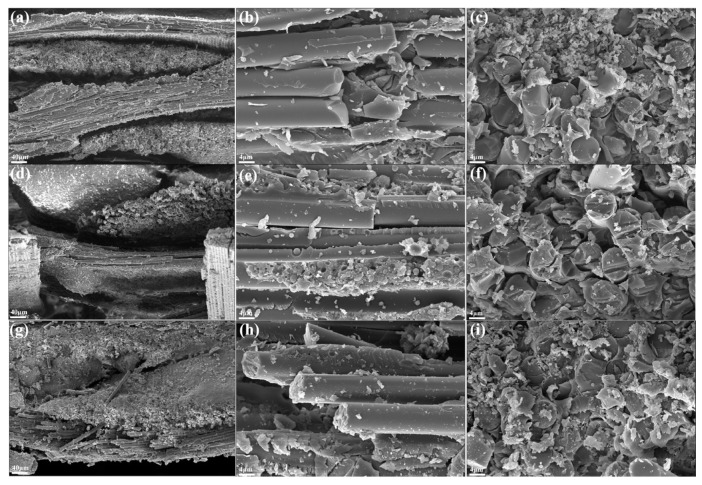
The SEM images of the flexural fracture surface of F_0_H_0_: (**a**–**c**), F_50_H_0_: (**d**–**f**), and F_50_H_10_: (**g**–**i**).

The flexural strength of the composites with different formulations is shown in Figure 8. The flexural strength of F_50_H_0_ was up to 452.4 MPa, which was 19.8% higher than that of F_0_H_0_, indicating that fused SiO_2_ could significantly improve the flexural strength of the composite. The particle size distribution of fused SiO_2_ is wide, varying from nanometers to micrometers, so it could exert a multi-scale synergistic reinforcing effect on the benzoxazine resin matrix (Figure 9). In addition, both fused SiO_2_ and the cured benzoxazine resin contained hydroxyl groups; thus, a strong intermolecular reaction was formed between the fused SiO_2_ and benzoxazine resin. Therefore, fused SiO_2_ had good compatibility with the benzoxazine resin and robust interfaces that were formed between them, which improved the mechanical strength (Figure 10). However, the flexural strength of the composites significantly decreased after introducing h-BN. When the dosage of h-BN exceeded 15 phf, the decrease in flexural strength was particularly significant. The flexural strength of F_50_H_20_ was decreased to 342.8 MPa, which was 9.2% lower than that of F_0_H_0_. This is possibly due to the chemically inert surface of h-BZ and the lack of interaction between h-BN and benzoxazine resin. The compatibility between h-BN and benzoxazine resin is poor; thus, the formation of the weak interface hinders the load from being effectively transferred and uniformly distributed, resulting in worse mechanical strength. Therefore, the addition of fused silica and an appropriate amount of h-BN can improve the bearing capacity of the composites. Notably, the flexural strength of the composites described in this work was much higher than those reported in previous works [14,27].

### 3.4. Oxyacetylene Ablation Behavior

**Figure 11:**
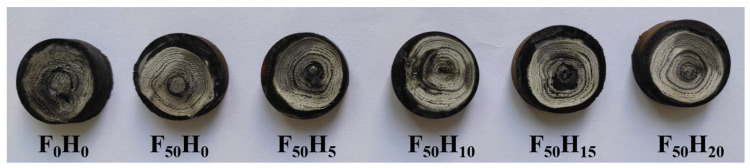
The morphology of the samples after oxyacetylene ablation.

**Figure 12:**
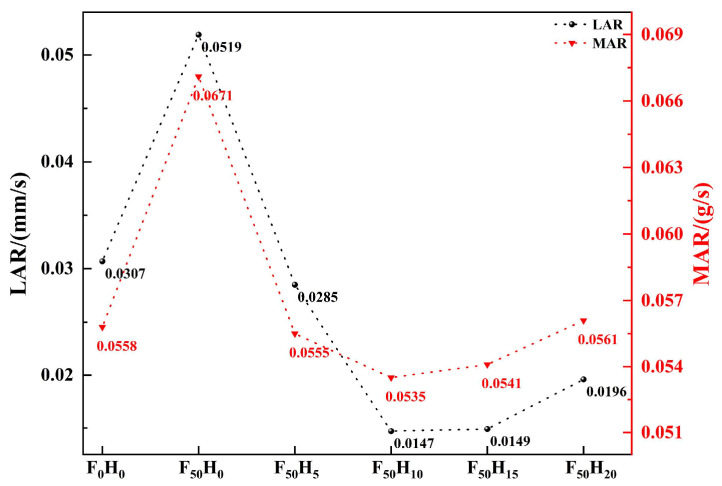
The LAR and MAR of the composites with different contents of fused SiO_2_ and h-BN.

**Figure 13:**
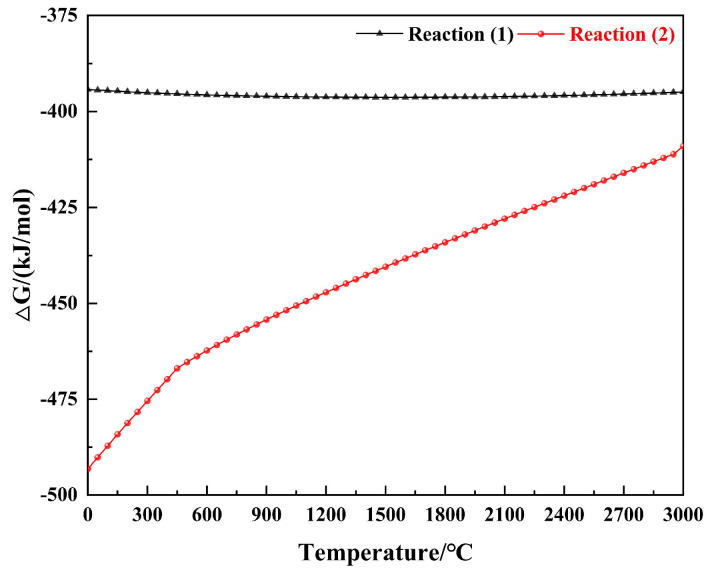
ΔG curves of the oxidation reactions.

Furthermore, the ablation resistance of the composites with different formulations was evaluated under an oxyacetylene flame, and the morphology of the samples after the oxyacetylene ablation is shown in Figure 11. There was a significant difference in the LAR of the composites with different contents of fused SiO_2_ and h-BN, indicating that the ceramizable fillers affected the ablation behavior (Figure 12). The LAR of F_0_H_0_ was 0.0307 mm/s, indicating a poor ablation resistance because the PyC was prone to oxidation at a high temperature, as confirmed by the change in Gibbs free energy (ΔG) (Figure 13). The LAR of F_50_H_0_ increased to 0.0519 mm/s, indicating that the ablation resistance of the composite became worse after the introduction of fused SiO_2_ alone. The increase in LAR was mainly due to the low melting point of the fused SiO_2_, which was easily peeled off under an oxyacetylene flame. After the introduction of h-BN, the LAR of the composite exhibited a dramatic change: the LAR first decreased and then increased with an increase in the h-BN content. Among them, the LAR of F_50_H_10_ exhibited the lowest value of 0.0147 mm/s, which was much lower than that reported in the previous works [21,26,27,28]. The LAR of F_50_H_20_ rebounded to 0.0196 mm/s. In addition, the MAR of the composites showed a trend similar to that of the LAR. The above results suggested that an appropriate amount of h-BN could significantly improve the ablation resistance of the composites.

C + O_2_ = CO_2_ (1)

In order to explore the reasons for the different ablation behaviors and ablation mechanisms, the morphology of the ablated surface and chemical bonding states were investigated.

**Figure 14:**
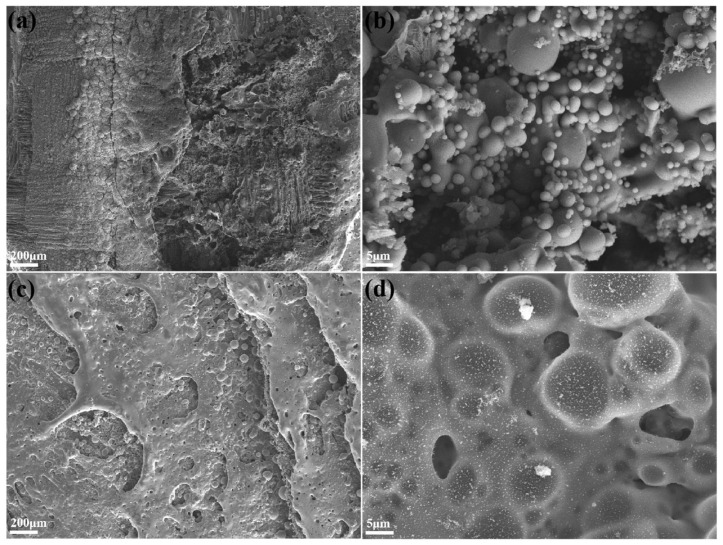
The micromorphology of the ablated surface in the central region: (**a**,**b**) F_0_H_0_ and (**c**,**d**) F_50_H_10_.

As depicted in Figure 14, there was a significant difference in the morphology of F_0_H_0_ and F_50_H_10_ after exposure to an oxyacetylene flame. The ablated surface of F_0_H_0_ was loose, with a number of pores and cracks appearing. The matrix underwent intense pyrolysis reactions under an oxyacetylene flame; thus, the PyC suffered from severe oxidization. Defects provided channels for the oxygen to penetrate the inner material, leading to severe thermochemical erosion. Moreover, a number of molten spheres were also observed, which were formed by the melting of SiO_2_ in quartz fibers. Due to the high surface energy, the molten SiO_2_ was difficult to spread on the surface of PyC. The adhesion between the molten spheres and PyC was relatively poor, making it prone to thermomechanical denudation, i.e., scouring and peeling off by the high-temperature and high-speed oxyacetylene flame.

On the contrary, the ablated surface of F_50_H_10_ was much denser and had fewer defects. It could be clearly observed that although there were molten spheres on the ablated surface of F_50_H_10_, they were bonded tightly together. This was due to the oxidation reaction of h-BN, where the generated B_2_O_3_ further reacted with SiO_2_ to form a relatively continuous borosilicate glass layer. The formed borosilicate glass could not only fill the defects formed by the pyrolysis of the matrix and oxidation of the PyC but also served as a thermal protective barrier to block oxygen, slowing down the erosion of the inner material. In addition, h-BN was more prone to oxidization than PyC. The preferential oxidation of h-BN can consume oxygen and delay the thermochemical erosion of the PyC. Moreover, the wettability and adhesion between the borosilicate glass and PyC yeilded better results, which was beneficial for improving their anti-scouring ability. As a result, F_50_H_10_ suffered weaker thermochemical erosion and thermomechanical denudation, which could explain its superior ablation resistance.

4/3BN+ O_2_ = 2/3B_2_O_3_ + 2/3N_2_ (2)

**Figure 15:**
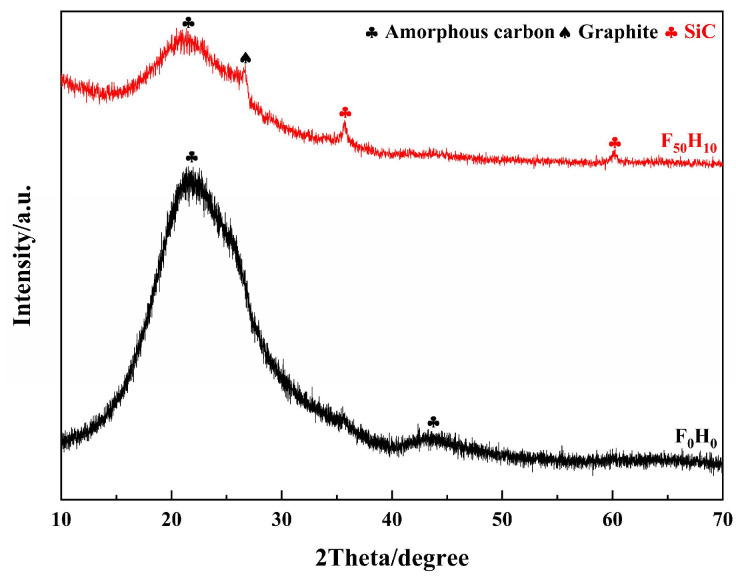
XRD patterns of the composites after the ablation test.

**Figure 16:**
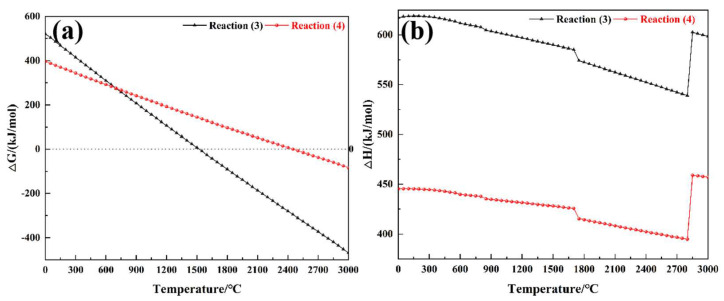
ΔG (**a**) and ΔH (**b**) of carbothermal reduction reactions.

**Figure 17:**
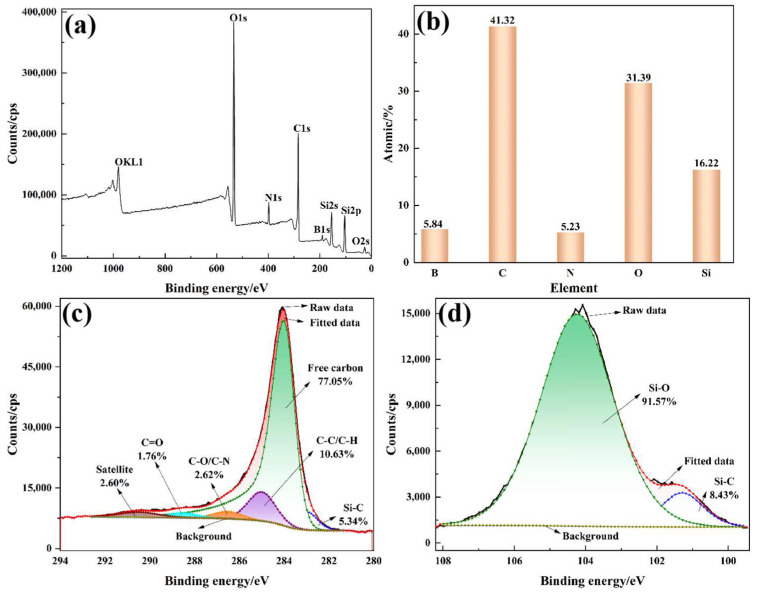
XPS survey spectrum (**a**), elemental contents (**b**), C1s spectrum (**c**), and Si2p spectrum (**d**).

Meanwhile, the structure of the composite materials after the ablation test was also identified using XRD (Figure 15). In the XRD pattern of the ablated F_0_H_0_, there were two broad amorphous humps at around 22° and 44°, which corresponded to PyC. On the contrary, several diffraction peaks appeared in the XRD pattern of the ablated F_50_H_10_. The diffraction peak at 26.7° was attributed to the (002) plane of graphite, whereas the diffraction peaks at 35.7° and 60.2° were derived from SiC. The PyC in F_0_H_0_ existed as an amorphous phase, whereas F_50_H_10_ contained a portion of graphite. Moreover, partial PyC in F_50_H_10_ was involved in carbothermal reduction reactions with SiO_2_ and was converted in situ into SiC, which acted as a refractory and antioxidant. Furthermore, the ΔG values of the aforementioned carbothermal reduction reactions were calculated (Figure 16). The ΔG values of Reaction (3) and (4) were below zero (especially Reaction (3)) under conditions of oxygen acetylene flame temperature (up to 3000 °C), suggesting that there was a great tendency for PyC to react with SiO_2_ spontaneously. Therefore, it was theoretically confirmed that the aforementioned carbothermal reduction reactions occurred. In addition, the chemical bonding states of the products in the ablation center region of F_50_H_10_ were characterized by XPS (Figure 17). The appearance of the Si 2p and C 1s peaks confirmed the existence of SiC in the sample, which was consistent with the XRD results.

SiO_2_ + 3C = SiC + 2CO (3)

SiO_2_ + 2C = SiC + CO_2_ (4)

A robust thermal protection barrier was formed on the surface of PyC, avoiding direct exposure to the oxyacetylene flame. Moreover, there was an intense endothermic effect during the carbothermal reduction reactions, as confirmed by the enthalpy change (ΔH) curves shown in Figure 16b, which alleviated the thermochemical erosion of PyC, leading to the superior ablation resistance of F_50_H_10_.

**Figure 18:**
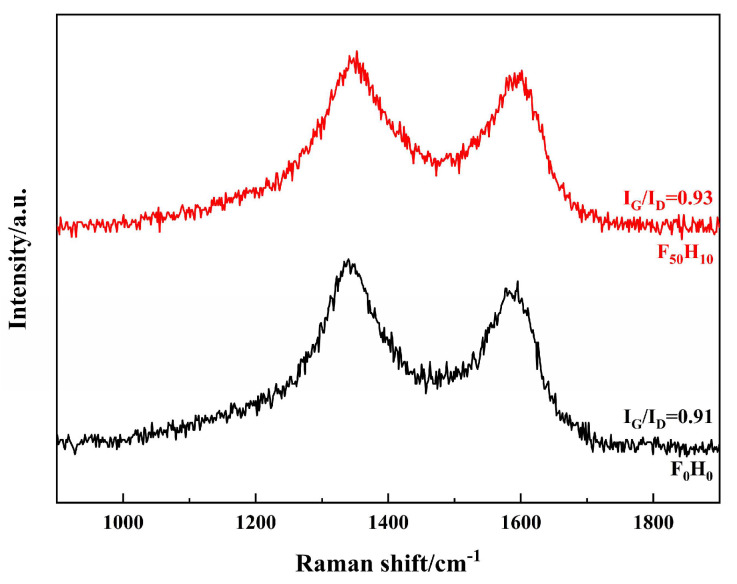
Raman spectra of the products in the central region of the ablated surface.

Raman spectra of the ablated surface were recorded, and the degree of graphitization was calculated. The peak at around 1600 cm^−1^ was called the G band, corresponding to an ideal graphite lattice, whereas the peak at around 1350 cm^−1^ was called the D band, corresponding to a graphite lattice with defects. Therefore, the intensity ratio of the G band to the D band (I_G_/I_D_) can be used to estimate defects in carbon materials [8,20]. A larger I_G_/I_D_ value indicates fewer defects and a higher degree of graphitization. As shown in Figure 18, the I_G_/I_D_ value of the products in the ablation center region of F_50_H_10_ was slightly higher than that of F_0_H_0_, suggesting that the degree of graphitization of PyC increased after introducing the ceramizable fillers.

### 3.5. Ablation Mechanisms

**Figure 19:**
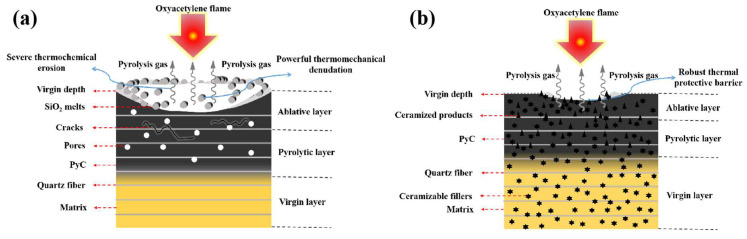
Schematic of the ablation mechanisms of F_0_H_0_ (**a**) and F_50_H_10_ (**b**).

Furthermore, possible ablation mechanisms were revealed based on the aforementioned results and discussion (Figure 19). In the ablated F_0_H_0_ sample, the matrix underwent violent pyrolysis reactions, and the resulting PyC was severely oxidized. Thus, it suffered from severe thermochemical erosion. The quartz fibers melted under the oxyacetylene flame, but the melted SiO_2_ failed to spread on the PyC; thus, they were easily peeled off by the high-temperature and high-speed oxyacetylene flame. F_0_H_0_ exhibited powerful thermomechanical denudation and poor ablation resistance.

In contrast, the preferential oxidation of h-BN occurred in F_50_H_10_, leading to oxygen consumption. Moreover, the generated B_2_O_3_ reacted with SiO_2_ to form a relatively continuous borosilicate glass layer, which acted as a self-healing agent and a thermal protective barrier. In addition, some PyC was involved in carbothermal reduction reactions with SiO_2_. The readily oxidized PyC and the SiO_2_ with a relatively low melting point were converted in situ into refractory carbide, which had an intense endothermic effect. The generated SiC served as a pinning phase and was embedded in the borosilicate glass. Therefore, a robust thermal protective barrier with SiC as the skeleton and borosilicate glass as the matrix was constructed on the surface of PyC. As a result, F_50_H_10_ underwent less thermochemical erosion and thermomechanical denudation and exhibited satisfactory ablation resistance.

## 4. Conclusions

In this work, a novel ceramizable composite of quartz fiber/benzoxazine resin modified with fused SiO_2_ and h-BN was prepared. The obtained ceramizable composite with an appropriate amount of fused SiO_2_ and h-BN added (e.g., F_50_H_10_) had excellent thermal stability, flexural strength, and ablation resistance.

The peak degradation temperature and the residue yield heated at 1400 °C in the F_50_H_10_ sample were 533.2 °C and 71.5%, respectively, which were much higher than those of the pristine quartz fiber/benzoxazine resin composite (F_0_H_0_). F_50_H_10_ achieved a flexural strength of 402.2 Mpa, indicating an excellent load-bearing capacity. After being exposed to an oxyacetylene torch at 4.2 MW/m^2^, its linear ablation rate was as low as 0.0147 mm/s, which was 52.1% less than that of the pristine quartz fiber/benzoxazine resin composite, demonstrating that the modification can significantly improve its ablation resistance. Moreover, the microstructure, phase structure, chemical bonding states, and degree of graphitization of the ablated composites were identified, and the ablation mechanisms were revealed. It was revealed that a robust thermal protective barrier with SiC as the skeleton and borosilicate glass as the matrix was constructed in situ, leading to superior ablation resistance.

## Figures and Tables

**Table 1:** Formulas of quartz fiber/benzoxazine resin composite incorporated with different contents of fused SiO_2_ and h-BN.

Samples	Benzoxazine Resin/g	Quartz Fiber/g	Fused SiO_2_/g	h-BN/g
F_0_H_0_	75	100	0	0
F_50_H_0_	75	100	50	0
F_50_H_5_	75	100	50	5
F_50_H_10_	75	100	50	10
F_50_H_15_	75	100	50	15
F_50_H_20_	75	100	50	20

**Table 2:** Detailed test conditions of the oxygen–acetylene ablation test.

Parameters	Values	Parameters	Values
Total unburned gas flow/(L/h)	2628	Diameter of the nozzle tip/(mm)	2
Oxygen gas flow/(L/h)	1512	Distance between nozzle tip and sample surface/(mm)	10
Acetylene gas flow/(L/h)	1116	Flame ablation angle/(°)	90
Oxygen pressure/(MPa)	0.4	Heat flux/(kW/m^2^)	4328
Acetylene pressure/(MPa)	0.095	Temperature of ablation flame/(°C)	3000 ± 300

**Table 3:** Characteristic absorption bands of samples.

Wave Number/cm^−1^	Vibrational Mode
935	Bending vibration of out-of-plane C-H on the oxazine ring
1035.1224	Symmetric and asymmetric stretching vibration of C-O-C on the oxazine ring
~3420	Stretching vibration of O-H of phenols
1148.1363	Symmetric and asymmetric stretching vibration of C-N-C on the oxazine ring
~1110	Stretching vibration of C-N-C

## Data Availability

The data presented in this study are available upon request from the corresponding author.

## Supplementary Materials

The following supporting information can be downloaded at: https://www.mdpi.com/article/10.3390/polym15224430/s1, Table S1: The main parameters for the materials that were utilized; Table S2: Degradation products of F0H0 in the gas phase; Table S3: Degradation products of F50H0 in the gas phase; Table S4: Degradation products of F50H10 in the gas phase.
