# Supplementary material for: A Novel Fused SiO2 and h-BN Modified Quartz Fiber/Benzoxazine Resin Ceramizable Composite with Excellent Flexural Strength and Ablation Resistance
Source: Polymers (Basel). 2023 Nov 16;15(22):4430. doi: 10.3390/polym15224430 (PMC10675632; doi:10.3390/polym15224430)
Supplement: Supplementary file 1 [file polymers-15-04430-s001.zip › polymers-2666429-SI.pdf]

A novel fused SiO_2 and h-BN modified quartz fiber/benzoxazine resin ceramizable composite with excellent flexural strength and ablation resistance

Zongyi Deng [1, 2], Yunfei Lv [3], Minxian Shi [1], Zhixiong Huang [a, b], Wenchao Huang [1 *]

[1] Key laboratory of Advanced Technology for Specially Functional Materials, Ministry of Education, Wuhan University of Technology, Wuhan, 430070, China

[2] Hubei Longzhong Laboratory, Xiangyang, 441000, China

[3] Beijing FRP Institute Composite Materials Co., Ltd., Beijing, 102101, China

1. Supplementary tables.

Table S1 The main parameters for the used materials.

Materials	Specifications	Density/(g/cm^3)	Thermal conductivity (20 °C)/(W/m^{-1}·K^{-1})
Benzoxazine resin	CB6800	1.20	0.18
Quartz fiber plain cloth	B type	2.2	1.40
Fused SiO_2 particles	1 μm, 99.5%	2.2 (True density)	1.38
h-BN particles	1 μm, 99.5%	2.27 (True density)/ 1.11 (Bulk density)	~550 (In-plane)

Table S2 Degradation products of F_0H_0 in the gas phase.

No.	Retention time/min	Component	Structural formula	Chemical formula	M_w	Content/ %
1	3.986	Cyclopentanone	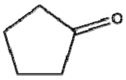	C_5H_8O	84.12	1.06
2	4.626	2-Furanmethanamine	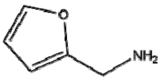	C_5H_7NO	97.12	3.76
3	5.163	1H-Pyrrole, 2,5-dimethyl-	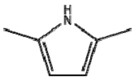	C_6H_9N	95.15	0.88
4	5.612	1H-Pyrrole-2-carboxaldehyde, 1-methyl-	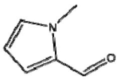	C_6H_7NO	109.13	1.75
5	6.615	Ethanol, 2,2'-oxybis-	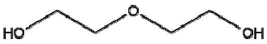	$C_4H_{10}O_3$	106.12	1.09
6	6.910	Phenol	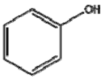	C_6H_6O	94.11	6.13
7	7.221	Benzofuran	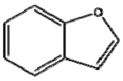	C_8H_6O	118.14	0.83
8	7.671	1-Hexanol, 2-ethyl-	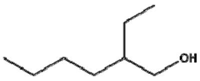	$C_8H_{18}O$	130.23	1.44
9	8.086	Phenol, 2-methyl-	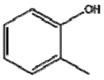	C_7H_8O	108.14	9.11
10	8.397	p-Cresol	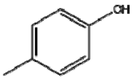	C_7H_8O	108.14	1.87
11	8.795	3-Pyridinol	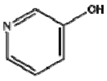	C_5H_5NO	95.1	0.74
12	8.864	Nonanal	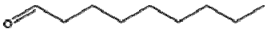	$C_9H_{18}O$	142.24	0.67
13	8.933	Phenol, 2,6-dimethyl-	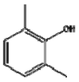	$C_8H_{10}O$	122.17	3.04
14	9.002	Benzofuran, 2-methyl-	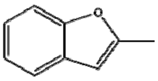	C_9H_8O	132.16	1.11
15	9.539	Phenol, 2,4-dimethyl-	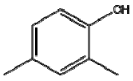	$C_8H_{10}O$	122.17	3.75
16	10.403	Phenol, 2,3,5-trimethyl-	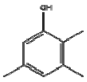	$C_9H_{12}O$	136.19	1.73
17	10.888	2-Coumaranone	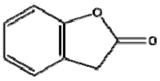	$C_8H_6O_2$	134.13	1.17
18	11.458	Furan, 3-phenyl-	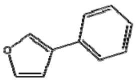	$C_{10}H_8O$	144.17	0.63
19	12.150	2(3H)-Benzofuranone, 3-methyl-	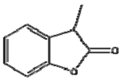	$C_9H_9O_2$	148.16	0.37
20	12.514	Benzene, 2,4-diisocyanato-1-methyl-	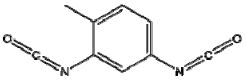	$C_9H_6N_2O_2$	174.16	7.05
21	12.981	1,3-Benzenediamine, 2-methyl-	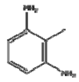	$C_7H_{10}N_2$	122.17	0.66

22	13.136	7-Hydroxy-1-indanone	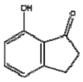	$C_9H_8O_2$	148.16	8.00
23	13.257	2-Methyl-5,6,7,8-tetrahydroquinoxaline	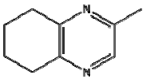	$C_9H_{12}N_2$	148.21	10.68
24	13.482	1H-Isoindole-1,3(2H)-dione, 2-methyl-	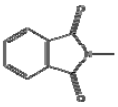	$C_9H_7NO_2$	161.16	1.35
25	14.330	Benzenemethanol, .alpha.-1-pentynyl	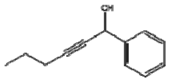	$C_{12}H_{14}O$	174.24	1.17
26	15.264	Naphthalene, 2,3-dimethoxy-	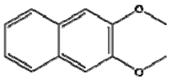	$C_{12}H_{12}O_2$	188.23	2.30
27	15.437	Coumarin, 5,7,8-trimethyl-	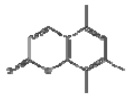	$C_{12}H_{12}O_2$	188.23	0.40
28	15.921	2,4(1H,3H)-Pyrimidinedione, 6-methyl-1-phenyl-	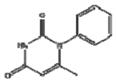	$C_{11}H_{10}N_2O_2$	202.21	1.37
29	16.094	4H-1-Benzopyran, 4,4,5,8-tetramethyl-	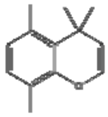	$C_{13}H_{16}O$	188.27	1.91
30	16.319	1,3,5-Trimethyl-2-cyclohexylbenzene	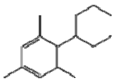	$C_{15}H_{22}$	202.34	0.97
31	16.924	Cyclohexanone, 2-[(4-methoxyphenyl)methylene]-	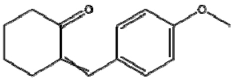	$C_{14}H_{16}O_2$	216.28	0.97
32	19.225	1,3-Diamino-5,6-dihydro-7-methylbenzo[f]quinazoline	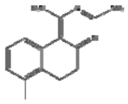	$C_{13}H_{14}N_4$	226.28	2.37
33	19.346	n-Hexadecanoic acid	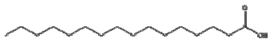	$C_{16}H_{32}O_2$	256.43	1.50
34	19.467	Dibutyl phthalate	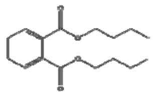	$C_{16}H_{22}O_4$	278.35	0.50
35	21.490	Phenol, 4,4'-(1-methylethylidene)bis-	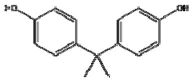	$C_{15}H_{16}O_2$	228.29	0.67
36	24.500	Bis(2-ethylhexyl) phthalate	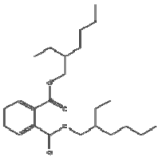	$C_{24}H_{38}O_4$	390.56	15.64

Table S3 Degradation products of $F_{50}H_{0}$ in the gas phase.

No.	Retention time/min	Component	Structural formula	Chemical formula	M_w	Content/ %
1	3.986	Cyclopentanone	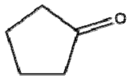	C_5H_8O	84.12	0.64
2	4.678	1H-Pyrrole, 3-methyl-	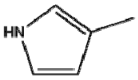	C_5H_7N	81.12	1.75
3	5.162	1H-Pyrrole, 2,5-dimethyl-	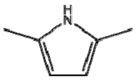	C_6H_9N	95.15	0.55
4	5.612	1H-Pyrrole-2-carboxaldehyde, 1-methyl-	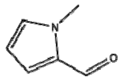	C_6H_7NO	109.13	0.91
5	6.235	3-Pyridinol, 2,6-dimethyl-	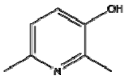	C_7H_9NO	123.16	0.26
6	6.892	Phenol	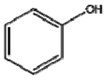	C_6H_6O	94.11	5.07
7	7.221	Benzofuran	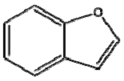	C_8H_6O	118.14	0.53
8	7.670	1-Hexanol, 2-ethyl-	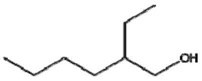	$C_8H_{18}O$	130.23	0.72
9	8.068	Phenol, 2-methyl-	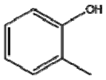	C_7H_8O	108.14	7.43
10	8.397	p-Cresol	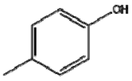	C_7H_8O	108.14	1.77
11	8.795	3-Pyridinol	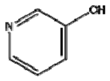	C_5H_5NO	95.1	0.43
12	8.933	Phenol, 2,6-dimethyl-	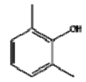	$C_8H_{10}O$	122.17	2.92
13	9.002	Benzofuran, 2-methyl-	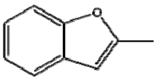	C_9H_8O	132.16	0.73
14	9.538	Phenol, 2,4-dimethyl-	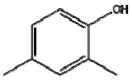	$C_8H_{10}O$	122.17	3.09
16	10.109	1H-Pyrrole, 1-(2-furanylmethyl)-	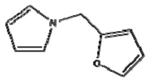	C_9H_9NO	147.18	0.19
16	10.196	2-Allylphenol	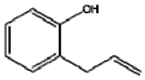	$C_9H_{10}O$	134.18	0.49
17	10.403	Phenol, 2,3,5-trimethyl-	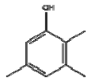	$C_9H_{12}O$	136.19	1.09
18	10.507	1H-Benzimidazole, 5,6-dimethyl-	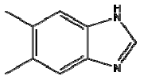	$C_9H_{10}N_2$	146.19	0.41
19	10.801	Benzenamine, N,N,2-trimethyl-	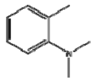	$C_9H_{13}N$	135.21	0.15
20	10.870	2-Coumaranone	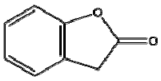	$C_8H_6O_2$	134.13	0.95

21	11.130	Hydrocoumarin	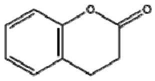	$C_9H_8O_2$	148.16	0.19
22	11.441	Furan, 3-phenyl-	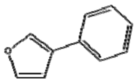	$C_{10}H_8O$	144.17	0.46
23	12.029	1-Naphthalenol, 4-methyl-	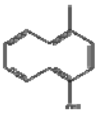	$C_{11}H_{10}O$	158.2	0.57
24	12.150	2(3H)-Benzofuranone, 3-methyl-	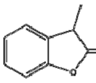	$C_9H_9O_2$	148.16	0.37
25	12.513	Benzene, 2,4-diisocyanato-1-methyl-	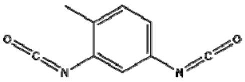	$C_9H_6N_2O_2$	174.16	1.84
26	13.136	7-Hydroxy-1-indanone	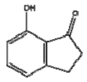	$C_9H_8O_2$	148.16	1.88
27	13.205	2H-Benzimidazol-2-one, 1,3-dihydro-5-methyl-	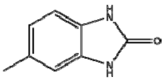	$C_8H_8N_2O$	148.17	1.73
28	13.465	1H-Isoindole-1,3(2H)-dione, 2-methyl-	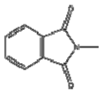	$C_9H_7NO_2$	161.16	1.35
29	13.914	Phthalimide	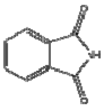	$C_8H_5NO_2$	147.13	0.23
30	14.329	Benzenemethanol, .alpha.-1-pentynyl	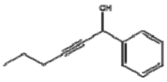	$C_{12}H_{14}O$	174.24	0.92
31	14.969	Coumarin, 4,5,7-trimethyl-	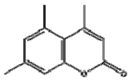	$C_{12}H_{12}O_2$	188.23	0.45
32	15.264	Naphthalene, 2,3-dimethoxy-	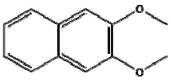	$C_{12}H_{12}O_2$	188.23	1.85
33	15.436	Coumarin, 5,7,8-trimethyl-	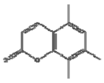	$C_{12}H_{12}O_2$	188.23	0.30
34	15.921	2,4(1H,3H)-Pyrimidinedione, 6-methyl-1-phenyl-	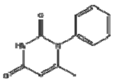	$C_{11}H_{10}N_2O_2$	202.21	1.13
35	16.076	4H-1-Benzopyran, 4,4,5,8-tetramethyl-	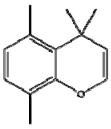	$C_{13}H_{16}O$	188.27	0.96
36	16.319	1,3,5-Trimethyl-2-cyclohexylbenzene	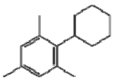	$C_{15}H_{22}$	202.34	1.25
37	16.872	Ethanone, 1-(1-hydroxy-2-naphthalenyl)-	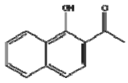	$C_{12}H_{10}O_2$	186.21	0.31
38	16.924	Cyclohexanone, 2-[(4-methoxyphenyl)methylene]-	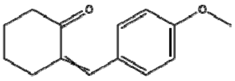	$C_{14}H_{16}O_2$	216.28	0.62
39	17.287	Tetradecanoic acid	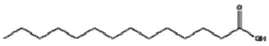	$C_{14}H_{28}O_2$	228.38	1.15
40	18.342	Pentadecanoic acid	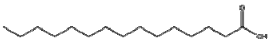	$C_{15}H_{30}O_2$	242.4	1.02
41	19.155	Palmitoleic acid	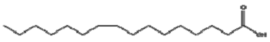	$C_{16}H_{30}O_2$	254.41	1.42

42	19.224	1,3-Diamino-5,6-dihydro-7-methylbenzo[f]quinazoline	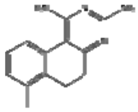	$C_{13}H_{14}N_4$	226.28	0.51
43	19.345	n-Hexadecanoic acid	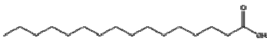	$C_{16}H_{32}O_2$	256.43	4.65
44	19.466	1,2-Benzenedicarboxylic acid, bis(2-methylpropyl) ester	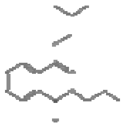	$C_{16}H_{22}O_4$	278.35	0.76
45	19.864	Phenol, 2-[(4-hydroxyphenyl)methyl]-	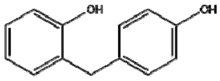	$C_{13}H_{12}O_2$	200.24	0.94
46	21.023	Oleic Acid	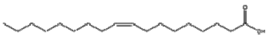	$C_{18}H_{34}O_2$	282.47	1.39
47	21.231	Octadecanoic acid	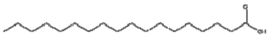	$C_{18}H_{36}O_2$	284.48	0.68
48	21.490	Phenol, 4,4'-(1-methylethylidene)bis-	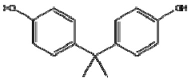	$C_{15}H_{16}O_2$	228.29	10.75
49	21.871	9,10-Anthracenedione, 1-ethyl-	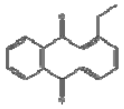	$C_{16}H_{12}O_2$	236.27	0.33
50	21.940	2-(4'-Hydroxyphenyl)-2-(4'-methoxyphenyl) propane	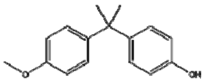	$C_{16}H_{18}O_2$	242.32	0.39
51	22.009	1,4-Benzenediamine, N-(1-methylethyl)-N'-phenyl-	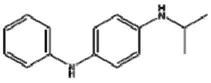	$C_{15}H_{18}N_2$	226.32	0.71
52	22.908	2,5-di-tert-Butyl-1,4-dimethoxybenzene	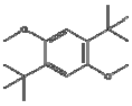	$C_{16}H_{26}O_2$	250.38	0.36
53	22.995	2,2'-(Alpha-methylbenzylidene)bis(5-methyl furan)	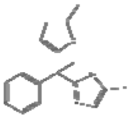	$C_{18}H_{18}O_2$	266.34	0.33
54	24.500	Bis(2-ethylhexyl) phthalate	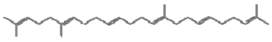	$C_{24}H_{38}O_4$	390.56	9.23
55	26.195	13-Docosenamide, (Z)-	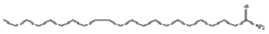	$C_{22}H_{43}NO$	337.59	2.48
56	26.541	Squalene	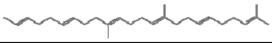	$C_{30}H_{50}$	410.73	12.19

Table S4 Degradation products of $F_{50}H_{10}$ in the gas phase.

No.	Retention time/min	Component	Structural formula	Chemical formula	M_w	Content /%
1	3.986	Cyclopentanone	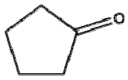	C_5H_8O	84.12	0.50
2	4.609	2-Furanmethanamine	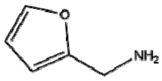	C_5H_7NO	97.12	6.88
3	5.162	1H-Pyrrole, 2,5-dimethyl-	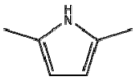	C_6H_9N	95.15	0.7
4	5.595	1H-Pyrrole-2-carboxaldehyde, 1-methyl-	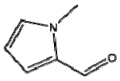	C_6H_7NO	109.13	2.56
5	6.909	Phenol	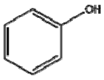	C_6H_6O	94.11	5.80
6	7.221	Benzofuran	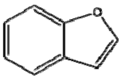	C_8H_6O	118.14	0.74
7	7.325	3-Thiophenecarboxaldehyde	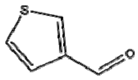	C_5H_4OS	112.15	0.65
8	7.670	1-Hexanol, 2-ethyl-	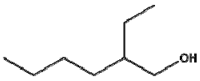	$C_8H_{18}O$	130.23	0.78
9	8.397	p-Cresol	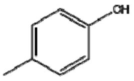	C_7H_8O	108.14	2.15
10	8.812	3-Pyridinol	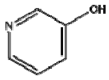	C_5H_5NO	95.1	1.17
11	8.933	Phenol, 2,6-dimethyl-	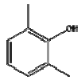	$C_8H_{10}O$	122.17	3.27
12	9.002	Benzofuran, 2-methyl-	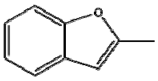	C_9H_8O	132.16	1.03
13	9.538	Phenol, 2,4-dimethyl-	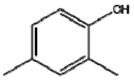	$C_8H_{10}O$	122.17	3.90
14	10.109	1H-Pyrrole, 1-(2-furanylmethyl)-	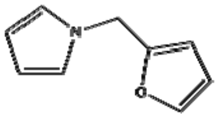	C_9H_9NO	147.18	0.41
15	10.334	4-Amino-1,6-dihydro-1-methyl-6-oxopyrimidine	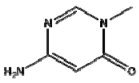	$C_5H_7N_3O$	125.13	0.51
16	10.403	Phenol, 2,3,5-trimethyl-	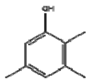	$C_9H_{12}O$	136.19	1.66
17	10.507	1H-Benzimidazole, 5,6-dimethyl-	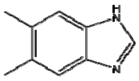	$C_9H_{10}N_2$	146.19	0.57
18	10.801	Benzenamine, N,N,2-trimethyl-	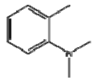	$C_9H_{13}N$	135.21	0.38
19	10.888	2-Coumaranone	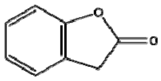	$C_8H_6O_2$	134.13	1.18

20	11.458	Furan, 3-phenyl-	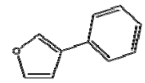	$C_{10}H_8O$	144.17	0.65
21	12.029	1-Naphthalenol, 4-methyl-	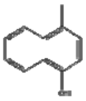	$C_{11}H_{10}O$	158.2	1.07
22	12.150	2(3H)-Benzofuranone, 3-methyl-	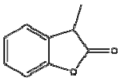	$C_9H_9O_2$	148.16	0.57
23	12.565	Cyclopentene, 4,4-dimethyl-	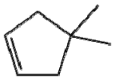	C_7H_{12}	96.17	0.55
24	13.119	N-(2-(furan-2-yl)vinyl)furan-2-amine	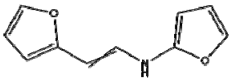	$C_{10}H_9NO_2$	175.19	5.33
25	13.482	1H-Isoindole-1,3(2H)-dione, 2-methyl-	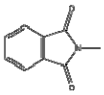	$C_9H_7NO_2$	161.16	1.04
26	13.724	Phenol, 3-amino-	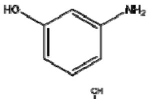	C_6H_7NO	109.13	0.56
27	14.330	Benzenemethanol, .alpha.-1-pentynyl	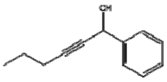	$C_{12}H_{14}O$	174.24	1.49
28	14.970	Coumarin, 4,5,7-trimethyl-	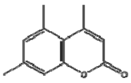	$C_{12}H_{12}O_2$	188.23	0.69
29	15.264	Naphthalene, 2,3-dimethoxy-	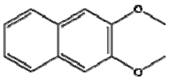	$C_{12}H_{12}O_2$	188.23	2.65
30	15.437	Coumarin, 5,7,8-trimethyl-	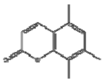	$C_{12}H_{12}O_2$	188.23	0.89
31	15.921	2,4(1H,3H)-Pyrimidinedione, 6-methyl-1-phenyl-	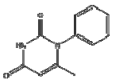	$C_{11}H_{10}N_2O_2$	202.21	1.88
32	16.077	4H-1-Benzopyran, 4,4,5,8-tetramethyl-	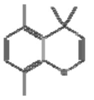	$C_{13}H_{16}O$	188.27	0.74
33	16.319	1,3,5-Trimethyl-2-cyclohexylbenzene	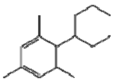	$C_{15}H_{22}$	202.34	0.91
34	16.924	Cyclohexanone, 2-[(4-methoxyphenyl)methylene]-	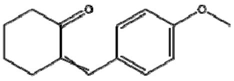	$C_{14}H_{16}O_2$	216.28	2.66
35	17.287	Tetradecanoic acid	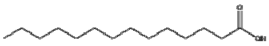	$C_{14}H_{28}O_2$	228.38	0.77
36	18.342	Pentadecanoic acid	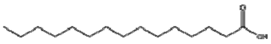	$C_{15}H_{30}O_2$	242.4	0.80
37	19.155	Palmitoleic acid	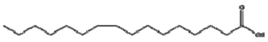	$C_{16}H_{30}O_2$	254.41	1.00
38	19.346	n-Hexadecanoic acid	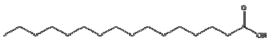	$C_{16}H_{32}O_2$	256.43	2.98
39	21.023	Oleic Acid	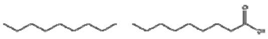	$C_{18}H_{34}O_2$	282.47	0.59
40	21.231	Octadecanoic acid	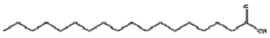	$C_{18}H_{36}O_2$	284.48	0.32
41	21.490	Phenol, 4,4'-(1-methylethylidene)bis-	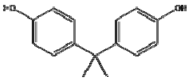	$C_{15}H_{16}O_2$	228.29	1.09

42	24.500	Bis(2-ethylhexyl) phthalate		$C_{24}H_{38}O_4$	390.56	18.75
43	26.541	Squalene	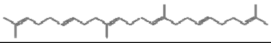	$C_{30}H_{50}$	410.73	6.18
